# Acute/Subacute and Sub-Chronic Oral Toxicity of a Hidroxytyrosol-Rich Virgin Olive Oil Extract

**DOI:** 10.3390/nu11092133

**Published:** 2019-09-06

**Authors:** Avilene Rodríguez-Lara, María Dolores Mesa, Jerónimo Aragón-Vela, Rafael A. Casuso, Cristina Casals Vázquez, Jesús M. Zúñiga, Jesús R. Huertas

**Affiliations:** 1 Department of Physiology, Institute of Nutrition and Food Technology “José Mataix”, Biomedical Research Center, University of Granada, Parque Tecnológico de la Salud, Avenida del Conocimiento s/n, 18016 Granada, Spain; 2 Department of Biochemistry and Molecular Biology II, Institute of Nutrition and Food Technology “José Mataix”, Biomedical Research Center, University of Granada, Parque Tecnológico de la Salud, Avenida del Conocimiento s/n, 18016 Granada, Spain; 3 Ibs.GRANADA. Biosanitary Research Institute of Granada, 18016 Granada, Spain; 4 Centro de Instrumentación Científica, University of Granada, 18016 Granada, Spain

**Keywords:** virgin olive oil, hydroxytyrosol, acute toxicity, subacute toxicity, subchronic toxicity, safety

## Abstract

The objective of this study was to determine the acute (one single dose), subacute (14 days), and sub-chronic (90 days) toxicity of an aqueous virgin olive oil (VOO) extract rich in hydroxytyrosol in rats. For acute/subacute toxicity, rats were divided into three groups. The control group received distilled water (*n* = 9), another experimental group received a single dose of 300 mg/kg (*n* = 3), and a third group received one dose of 2000 mg/kg (*n* = 4) during 14 days. The sub-chronic study included 60rats distributed in three groups (*n* = 20: 10 males and 10 females) receiving daily different three doses of the VOO extract in the drinking water during 90 days: (1) 100 mg/kg, (2) 300 mg/kg, and (3) 1000 mg/kg. In parallel, a fourth additional group (*n* = 20: 10 males and 10 females) did not receive any extract (control group). Clinical signs, body weight, functional observations of sensory and motor reactivity, hematological and biochemical analyses, and macroscopic and microscopic histopathology were evaluated. No adverse effects were observed after the administration of the different doses of the hydroxytyrosol-rich VOO extract, which suggests that the enrichment of VOO in its phenolic compound is safe, and can be used as functional foods for the treatment of chronic degenerative diseases.

## 1. Introduction

**Figure 1:**
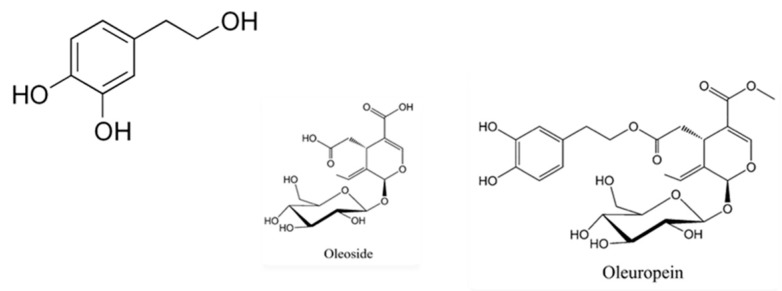
Chemical structure of hydroxytyrosol and derivatives.

There is clinical evidence that the Mediterranean diet is associated with beneficial health effects, in terms of morbidity and mortality [1], and is highly recommended for cardiovascular risk patients [2]. Virgin olive oil (VOO) is the main fatty source and is responsible for many of the health benefits attributed to this dietary pattern [3]. Both the presence of oleic acid and minor bioactive compounds may exert protective activities, but it seems that VOO polyphenols are the main bioactive components [4]. A systematic review has stated that consumption of VOO influences the expression of atherosclerotic-related genes at the moderate doses consumed within the Mediterranean diet. In addition, those authors concluded that hydroxytyrosol may exert changes in the expression of transcription factors involved in cell proliferation [5]. Hydroxytyrosol and its derivatives (Figure 1) are considered as the plant phenols exerting the most potent free radical scavenger activity that may protect blood lipids against oxidative damage. Anti-inflammatory and anti-platelet aggregation activities have been also attributed to hydroxytyrosol as responsible for its cardioprotective and neuroprotective properties [6]. In fact, the European Food Safety Authority (EFSA) approved two health claims regarding the antioxidant properties of VOO containing at least 5 mg of hydroxytyrosol and its related compounds (tyrosol and oleuropein) per 20 g [7]. Therefore, it is desirable to increase the polyphenol content of VOO in order to improve its benefits. In this way, previous studies have demonstrated the cardioprotective effect of VOO enriched in bioactive compounds from the olive oil in animals [8] and humans [9,10]. In fact, recent studies have shown a strong ergogenic effect on physical activity and remodeling of more efficient mitochondrial super-complexes with lower reactive oxygen-derived species production at doses 20 mg/kg and 300 mg/kg [11,12].

However, in terms of safety, although no adverse effects have been demonstrated until now, even at very high doses, there is little scientific evidence about the toxicological effects of hydroxytyrosol-rich VOOs [4]. The aim of this study was to evaluate the acute/subacute and subchronic toxicity of an aqueous VOO extract rich in hydroxytyrosol, according to the Organization for Economic Cooperation and Development (OECD) 408 regulation, in rats.

## 2. Materials and Methods

### 2.1. Hydroxytyrosol Isolation from a VOO Extract

The aqueous VOO extract rich in hydroxytyrosol was supplied by “EXTRACTS AND DERIVATES” (Granada, Spain), which is a company focused on the generation of bioactive extracts from the olive grove. The extract contained an initial concentration of 15% of hydroxytyrosol, which was confirmed by HPLC-UV (Agilent 1260 series) using a Hypersil Gold 5 μm chromatographic column (4.6 mm × 100 mm).

The VOO extract was dissolved and administered in the drinking water in order to minimize the manipulation of the animals. After calculating the median daily amount of water intake, dilutions of the extract were prepared in milli Q water to achieve final different doses of hydroxytyrosol: 100 mg/kg (low dose), 300 mg/kg (intermediate dose), and 1000 mg/kg (high dose). The doses were adjusted by the rat body weight every three days. Five hundred ml of the water enriched with the VOO extract were prepared every three days. To avoid possible oxidation of the extract by light, aluminum foil was used in the bottles.

### 2.2. Fine Tunning Study

First, we carried out a pilot study in order to set up the protocol of intervention. Three rats were maintained in metabolic individual cages, and food and water intake were monitored for five days. Then, the three rats were placed together in one cage, and food and water intake was also monitored for five days. In this initial study, we checked that the water intake of 37 mL/day was similar when the rats were placed independently or in groups of three animals.

### 2.3. Study Design

#### 2.3.1. Acute/Subacute Toxicity

According to the OECD Guidelines for Testing of Chemicals, acute/subacute toxicity was performed after administering an intermediate single dose of 300 mg/kg and a high dose of 2000 mg/kg for 14 days. The doses were as indicated in the OECD 420 protocol [13]. Eighteen Wistar SHD rats (HARLAN Ltd. Barcelona, Spain) that were three weeks old were used to perform the study. Before the start of the experimental period, the animals were kept in type III cages (1650 × 690 × 1870 cm) during the first five days for acclimatization. Animals were placed per cage and kept in modules with a sound level<325 lux, dark light cycles (12 h), temperature 20–24 °C, and humidity HR 50 + 10%.

In the first phase one group of rats (*n* = 3) received a single dose of 300 mg of hydroxytyrosol/kg by gavage using a gastric rigid cannula. The animals were observed for 24 h before the sacrifice. In the second phase, a second group of animals (*n* = 4) received a daily dose of 2000 mg/kg, that is considered as the maximum, during 14 days, which was given using a gastric rigid cannula. The control group (*n* = 9) received the same amount of liquid (i.e., water) by gavage for a period of 14 days. All the animals were fed the HARLAN Tekland diet *ad libitum*, which was provided 3–4 h after the administration of the VOO extract. The animals were observed daily during the 14 days of intervention before the sacrifice.

Body weight was controlled every two days [14] and toxic signs were monitored daily using the Irwin test (see below). Necropsy and the microscopic observation of fundamental organs and tissues (brain, cerebellum, oesophagus, spleen, stomach, liver, small intestine, large intestine, marrow, and kidney) were performed during the autopsy. Hematologicaland biochemical values were also measured.

#### 2.3.2. Sub Chronic Toxicity

Before the start of the experimental period, the animals were kept in type III cages (1650 × 690 × 1870 cm) for five days for acclimatization. Eighty Wistar SHD rats (HARLAN Ltd. Barcelona, Spain) at three weeks of age, were randomly divided into three intervention groups, and one control group (*n* = 20: 10 males and 10 females per group). The body weight intervals in the four groups were less than 20%. One group received a dose of 100 mg of hydroxytyrosol/kg/day in the drinking water, which is considered a low dose. The second group received a dose of 300 mg/kg/day in the drinking water, which is considered the intermediate dose. The third group received a dose of 1000 mg/kg/day in the drinking water, which was considered the high dose. The fourth control group only received drinking water without the VOO extract.

As determined in the pilot study, three animals were placed per cage and kept in modules with sound level<65 dB, air renewal 15–17/h, light <325 lux, dark light cycles (12 h), temperature 20–24 °C, and humidity HR 50 + 10%. The study protocol was based on the provisions of Directive 92/69/CEEE-OECD 408, according to the OECD-408 Guidelines for Testing of Chemicals, N° 408, entitled “Repeated Dose 90-Day Oral Toxicity Study in Rodents” [13].

All experiments were performed according to a protocol approved by the Institutional Animal Care and Use Committee of the University of Granada (procedures Granada, Spain. n°: 28/06/2016/116) and in accordance with the European Convention for the Protection of Vertebrate Animals used for Experimental and Other Scientific Purposes (CETS # 123), directive 2010/63/EU for the protection of animals used.

The consumption of the VOO extract was measured every three days. The intake of water with the VOO extract was calculated by the difference between a fixed amount prepared (500 mL/bottle) and the remaining volume, divided by the three rats per cuvette. Each time the data was recorded, a new bottle was freshly replenished. All animals received food *ad libitum*.

A clinical evaluation (Irwin test) was performed in all animals twice a day, between 8:00–9:00 a.m. in the morning, and 19:00–21:00 p.m. in the afternoon. The animals were weighed twice a week with a scale up to0.1 g [15].

### 2.4. Clinical Evaluation (Irwin Test)

To evaluate the reaction to intervention during the acute, sub-acute, and sub-chronic studies, each rat was analyzed daily for visible signs of morbidity and mortality, according to the Irwin test. Toxicity signs measured were perception (alert, stereotypy, passivity), mood (neat, restless, irritable-aggressive), motor activity (spontaneity, reactivity, tactile response), central nervous system (CNS) excitation and depression (tail raised, tremors, and convulsions), motor coordination (somersault recovery), diarrhea, salivation, skin and fur changes, and eye mucosa modifications. These parameters were evaluated during the 90 days of intervention based on OECD guidelines [14].

### 2.5. Hematological and Biochemical Analyses

At the end of the experimental period, blood samples were extracted from the cardiac puncture with previous inhalation anesthesia using isoflourane, in a mobile anesthesia equipment CA-EAC22, CIBERTEC (Madrid Spain). One mL of blood was collected in an EDTA-coated tube, stirred slightly, and kept at room temperature up to a maximum of 3 to 4 h for hematological analyses. The remaining blood (2–3 mL) was collected with heparin, centrifuged at 3000 rpm, and the separated plasma was frozen at −80 °C immediately for biochemical analysis.

Hematological parameters were determined in blood samples using a Mythic 22 CT C2 Diagnostic Analyzer (Orphée, SA, Switzerland). Total red blood cell count (RBC), hemoglobin, the percentage of total blood volume occupied by red blood cells (packed red blood cell volume, PCV), mean corpuscular volume (MCV), mean corpuscular hemoglobin (MCH), mean corpuscular hemoglobin concentration (MCHC), and red blood cell size distribution (RDW) were determined as erythrocyte indices. Total white blood cell count (WBC), differentiated neutrophils (NEU), monocytes (MON), lymphocytes (LYM), eosinophils (EOS), and basophils (BAS) were determined as total counts and percentages. Platelet total count (PLT), mean platelet volume (MPV), the percentage of total blood volume occupied by platelets (HTC), and platelet distribution width (PDW) were determined as platelet indices.

Plasma biochemical parameters include glucose, total cholesterol, triacylglycerides, total proteins, albumin, aspartate aminotransaminase (AST), alanine amino-transaminase (ALT), total bilirubin, urea, creatinine, sodium, potassium, chloride, phosphorus, and creatinin kinase isoenzyme MB (CK-MB), which were analyzed in a BS-200 Chemistry Analyzer (Shenzhen Mindra y Biomedical Electronics, Shenzhen, China). Globulins were calculated as the difference between total proteins and albumin, and the ratio albumin/globulins was also calculated.

### 2.6. Histopathological Examination

After bleeding, anesthetized animals were sacrificed with the intraperitoneal injection of sodium pentobarbital (30–40 mg/kg body weight) for subsequent necropsy. Necropsy was carried out according to Feldman et al. criteria [15]. The rats were examined for external abnormalities carefully. Then, different organs were extracted and macroscopic appearance was evaluated. Biopsies were studied in 14 different organs or systems, including liver, kidney, cerebellum, heart, esophagus, salivary gland, gonads, bone, bone marrow, nerve, pancreas, skin, windpipe, and thymus. For biopsies, tissue samples were preserved in 10% buffered formalin flasks and included inparaffin blocks. Rotary microtome sections were made in each block, and hematoxylin and eosin were used for staining before microscopic examination.

### 2.7. Statistical Analysis

Data are presented as the mean values *±* standard error of the mean (SEM). Theone-way analysis of variance (ANOVA) was used to compare mean values. Previously, the normality of the variables was analyzed by the Kolmogorov-Smirnov test, and the homogeneity of the variance was analyzed by the Levene test.

The differences between groups were established using the post-hoc Bonferroni test for homogeneous variances and Tamhane T2 for non-homogeneous variancevariables (chloride, albumin, phosphorous, AST, ALT). The outliers for each intervention were removed if kurtosis>1 and asymmetry>1 in the distribution of the responses. In all cases, more than 80% of the data were analyzed. All analyses were performed on an intention-to-treat basis. A *p* < 0.05 value was considered significant. A statistical package for the social sciences version 20 software was used to perform the statistical analysis (SPSS Inc., Chicago, IL, USA).

## 3. Results

### 3.1. Acute/Subacute Oral ToxicityWeight Evolution during the 14 Days of Intervention

Body weight gain was continuous without significant differences between the animals of the control group and the animals supplemented with the two doses of the VOO extract for the 14 days.

### 3.2. Sub Chronic Oral Toxicity Water Consumption and Weight Evolution during the 90 Days of Intervention

In the fine-tuning study, we confirmed the suitability of placing three rats per cage compared with maintaining only one rat per cage. Daily water consumption was calculated (37 mL/day), and was similar when three animals were placed together to those determined when rats were placed individually.

No significant differences were observed related to water consumption between the four groups of intervention. The amounts were 33 ± 0.19 mL/day for the control rats, and 38 ± 0.17 mL/day, 35 ± 0.27 mL/day, and 31 ± 0.46 mL/day for the rats supplemented with 100, 300, and 1000 mg/kg/day, respectively. During the first two weeks of the study, the rats receiving the high dose of the VOO extract drank 25% less water than the rest of the groups (non-significant). This difference was normalized in the following weeks of intervention, which showsan organoleptic acceptance of the VOO extract.

**Figure 2:**
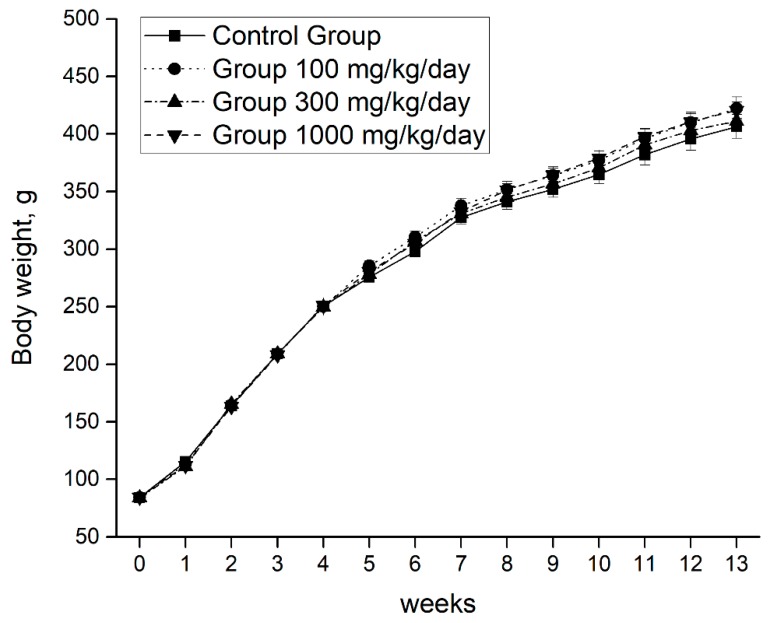
Body weight evolution during the 90 days of intervention. Data are means ± SEM. One-way ANOVA test and Bonferroni post hoc were used to compare results between groups at each time point. *P* < 0.05 was considered significant. One-way analysis of variance, ANOVA.

The growth was normal and continuous without significant differences between the individuals of the control group and the three intervention groups (Figure 2).

### 3.3. Clinical Evaluation: Irwin Test

#### 3.3.1. Acute/Subacute Oral Toxicity

During the intervention period of 14 days, we have not observed tremors, convulsions, numbness, salivation, or diarrhea. We also have not detected alterations in the skin or the hair of the eyes. The color of the urine was normal during handling in all cases.

#### 3.3.2. Sub Chronic Oral Toxicity

The survival ratio of the animals at the end of the experiment was 100%. The color of the hair was normal in all animals. Accidental cases of small alopecia were observed in four rats (two rats from the group supplemented with the low dose (100 mg/kg/day) and two rats from the group supplemented with the intermediate dose (300 mg/kg/day). In those four animals, no fungi were observed by means of Wood’s lamp, but a topical treatment with fungizel (tolnaftate, lindane, and benzoic acid, administered twice) provoked the recovery of the bald areas after 15 days. The rest of the animals presented a normal skin and fur aspect during the 90 days of study. None altered clinical sign of diarrhea, salivation, eye mucosa alteration, central nervous system stimulation or depression, or the way of walking (with the abdomen movingbackwards) were observed in the 80 animals of the experimental groups, including the control group and the three VOO extract-treated groups, during the intervention period. In addition, no trembling, convulsions, or numbness of limbs were observed. In addition, the color of the urine was normal in all animals.

### 3.4. Hematological and Biochemical Biomarkers

#### 3.4.1. Acute/Subacute Hematological Biomarkers

Hematological data of acute/subacute oral toxicity is indicated in Table 1. All hematological biomarkers were within the normal range at the end of the study. The RBC count and Hemoglobin were higher in the group supplemented with the maximum dose of the VOO extracts than in the animals supplemented with the low dose and control. PCV% and MON count were higher in the group supplemented with the low dose than in the high dose and the control group. The rest of the hematological parameters were similar in all groups.

#### 3.4.2. Sub Chronic Hematological Biomarkers

Hematological data are indicated in Table 2. All hematological biomarkers were within the normal ranges at the end of the experimental time in all groups. RBC count, PCV, and MCV were lower in the group supplemented with the low dose of the VOO extract than in animals supplemented the high dose. MCH was higher in the group supplemented with the high dose than in the control and intermediate-dose supplemented groups, while MCHC was lower in the high-dose group than in the other groups. Total and differentiated WBC count and percentages were similar in all groups of animals except the percentage of neutrophils that was lower in the high-dose group than in the other groups. Platelets count and MPV were higher in the control group than in the group receiving 100 mg/kg/day, while MPV was lower in the control group versus the supplemented groups, and higher in the supplemented low dose. The rest of hematological parameters were similar in all groups.

#### 3.4.3. Acute/Subacute Biochemical Biomarkers

Biochemical values are included in Table 3. CK-MB was higher in the group supplemented with the low dose (300 mg/kg) of the VOO extract if compared with the high-dose and with the control group. The rest of the biochemical parameters were similar between groups.

#### 3.4.4. Sub Chronic Biochemical Biomarkers

Biochemical values after 90 days of administration of the VOO extract are included in Table 4. Glucose plasma levels were lower in the group supplemented with the low dose of the VOO extract when compared with the high-dose supplemented group. Total protein plasma levels were lower in the group supplemented with the intermediate dose of the VOO extract compared with control group. Plasma bilirrubin was higher in the supplemented with the intermediate dose than in the control and high-dose supplemented groups. Sodium levels were higher in the supplemented groups when compared to the control group. The rest of the biochemical parameters were similar in all groups.

### 3.5. Histopathological Examination

#### 3.5.1. Acute/Subacute Oral Toxicity

**Figure 3:**
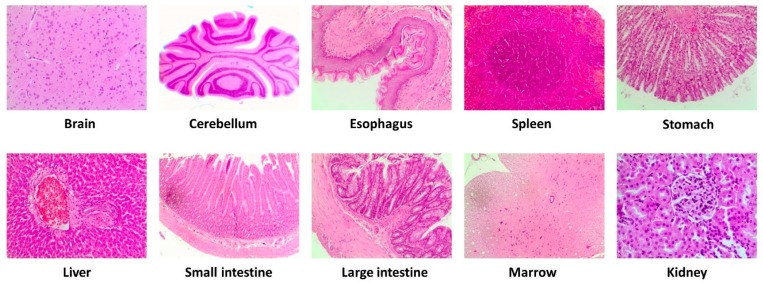
Representative microscopic evaluation (10×) of selected tissues after intervention with the high dose (2000 mg/kg/day) of a VOO extractin the study acute/subacute. Virgin olive oil (VOO).

The autopsy did not show any macroscopoic (i.e., color, size, or texture) or microscopic (Figure 3) alteration of the organs and tissues examined from the animals treated with the VOO extract compared with the control animals.

#### 3.5.2. Sub Chronic Oral Toxicity

**Figure 4:**
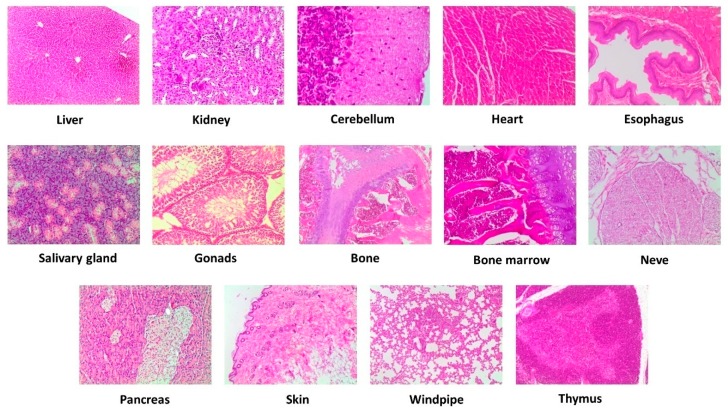
Representative microscopic evaluation (10×) of selected tissues after intervention with the high dose (1000 mg/kg/day) of a VOO extractin the study is sub chronic. Virgin olive oil, VOO.

Macroscopic examination of cerebellum, heart, esophagus, salivary gland, gonads, bone, bone marrow, nerve, pancreas, skin, windpipe, and thymus of VOO extract-treated rats revealed no abnormalities in the appearances (color, size, or texture) when compared with the control animals. No dose-dependent macro and microscopic histology were observed after an intervention with the three doses of the VOO extract when compared with the control animals. Microscopic evaluation exhibited no histological discordance, with a normal structure and absence of pathological lesions (Figure 4).

## 4. Discussion

VOO is the main fat source in the Mediterranean diet, and has been associated with the healthy benefits attributed to this dietary pattern [16]. The EFSA VOO-related health claims propose the effectiveness of VOO containing at least 5 mg of hydroxytyrosol and its related compounds (tyrosol and oleuropein) per 20 g [7]. Therefore, the enrichment of this oil with its own extracted bioactive compounds is the goal of the olive industry. However, it is necessary to prove the safety of VOO bioactive compound concentrates before they can be used for the enrichment of dietary oils or other foods, or for its nutraceutical use. In the present study, we demonstrated no toxic effect of a dose of 300 mg/kg/d of a VOO extract rich in hydroxytyrosol, in an acute single dose, subacute 14-days supplementation with a maximum dose (2000 mg/kg/d), and after the sub-chronic supplementation during 90 days with 100 mg/kg/d, 300 mg/kg/d and 1000 mg/kg/d, according to the OECD-408 guidelines.

Although minor hematological and biochemical differences were observed between the control and the VOO extract-supplemented groups, they did not follow a dose-dependent pattern, and all evaluated clinical, hematological, biochemical, and histological parameters were within normal ranges in all the animals, which indicatesno toxicological effect of this extract at the length and dosages examined. These results are of great interest, since they guarantee that the enrichment of VOOs or other nourishment with the VOO extract may be a good tool for the industrial preparation of functional foods that can be used as co-adjuvants for the prevention or treatment of chronic diseases such as cardiovascular, cancer, and other age-related diseases. Epidemiological evidence suggest that the phenols found in olive oil have anticancer properties and may play an important role in reducing inflammation, free radicals, and carcinogenesis as well as may improve the gut microbiota composition [17,18]. In addition, Borzi et al. [19] point out that olive oil polyphenols exert a protective effect against colorectal cancer. These studies highlight the need to investigate the doses at which polyphenols induce their health-related effects.

Therefore, a diet rich in polyphenols may be inversely associated with general mortality and CVD, certain types of cancer, cardiovascular diseases, anthropometric measures, and even mood [17]. The study of polyphenols is quite extensive, and it has investigated the metabolism and transformation of polyphenols into bioavailable molecules responsible for the preventive actions of polyphenols, including the transformation of carcinogens, modulation of the pathways responsible for cancer cell signalling, and cell cycle progression [18].

Little data has shownacute, subchronic, or chronic toxicity of VOO extracts [20]. The main component of the VOO extract is hydroxytyrosol and studies usually define the composition of the olive oil extract related to the content of this major pholyphenol. An acute toxicological study with an aqueous olive-pulp extract containing approximately 1400 mg/kg of pure hydroxytyrosol reported null mortality and morbidity [21]. In addition, these authors tested the toxic effect of a single dose of 5 g/kg, and established a lethal dose 50 (LD50) of around 3.5 g/kg of hydroxytyrosol. In addition, subchronic toxicity was evaluated at doses of 1000, 1500, and 2000 mg of hydoxytyrosol/kg, concluding no significant changes associated with the VOO extract compounds. Other authors have reported nosignificant toxic effects after a 90-days subchronic toxicity test of an extract with 35% of hydroxytyrosol, at doses of 125, 250, and 500 mg of hydroxytyrosol/kg/day [22,23], even though the last study reported a decrease in body weight and an increase in relative weight of liver, thymus, kidneys, and spleen in male rats. In addition, Kirkland et al. evaluated the acute toxicity of a hydroxytyrosol-enriched extract (40%) and determined that 2000 mg of hydroxytyrosol/kg was well tolerated.

Hydroxytyrosol has demonstrated antioxidant, anti-inflammatory, hypo-cholesterolemic, and pro-apoptotic activities [24] that may be responsible for the cardioprotective and anti-tumoral properties attributed to this molecule [25,26]. Few studies have evaluated the safety of pure hydroxytyrosol in animal studies following OECD-408 guidelines. Auñon-Calles et al. [27] evaluated the sub-chronic oral toxicological potential of pure hydroxytyrosol in rats. They reported that oral administration of hydroxytyrosol once a day during 90 days, at doses of 5, 50, and 500 mg/kg body weight, did not induce effects that could be considered of toxicological relevance. These authors proposed a No Observed Adverse Effects Level (NOAEL) of 500 mg/kg/d of pure hydroxytyrosol. Previously, D’Angelo et al. [28] found non-toxic effects after the administration of a single dose of 2 g/kg of body weight to rats. Furthermore, Martinez et al. [29] evaluated the acute 28-day oral toxicity of 2000 mg/kg/day of a hydroxytyrosol phosphatidyl-derivative, which shows no toxic effects compared with thecontrolanimals.

## 5. Conclusions

We conclude that oral administration of a VOO extract containing 15% of hydroxytyrosol did not induce effects that can be considered of toxicological relevance, and propose NOAEL dose of 1000 mg/kg/d of pure hydroxytyrosol.

## Figures and Tables

**Table 1:** Blood hematological parameters after 14 days of intervention with different doses of the VOO extract.

	Control	300 mg/kg (Intermediate Dose)	2000 mg/kg/d (Maximum Dose)	*p*
RBC (10^6^/µL)	7.05 ± 0.21 ^ab^	4.57 ± 1.26 ^a^	7.58 ± 0.21 ^b^	0.005
Hemoglobin (g/dL)	14.0 ± 0.4 ^a^	10.1 ± 2.7 ^b^	14.8 ± 0.3 ^ab^	0.029
PCV%	38.5 ± 1.2 ^a^	27.7 ± 7.8 ^b^	14.8 ± 0.3 ^ab^	0.033
MCV (fl)	54.63 ± 0.99	60.0 ± 5.65	53.87 ± 1.06	0.199
MCH	19.94 ± 0.38	22.16 ± 2.29	36.32 ± 0.28	0.167
MCHC (g Hb/dL)	36.53 ± 0.27	36.90 ± 0.81	36.32 ± 0.28	0.708
RDW	15.27 ± 1.14	15.0 ± 0.94	15.0 ± 0.94	0.511
WBC (10^6^/µL)	8.86 ± 0.82	6.13 ± 1.61	13.60 ± 4.47	0.146
LYM (10^6^/µL)	4.66 ± 0.49	4.03 ± 1.31	4.72 ± 0.46	0.804
MON (10^6^/µL)	0.11 ± 0.02	0.20 ± 0.05	0.12 ± 0.02	0.165
EOS (10^6^/µL)	0.11 ± 0.06	0.06 ± 0.03	0.07 ± 0.04	0.882
BAS(10^6^/µL)	0.17 ± 0.03	0.13 ± 0.03	0.25 ± 0.02	0.267
LYM%	83.8 ± 0.8	85.9 ± 2.2	78.2 ± 5.3	0.190
MON%	1.9 ± 0.1 ^a^	3.5 ± 0.3 ^b^	2.3 ± 0.4 ^ab^	0.017
EOS%	2.14 ± 0.91	1.43 ± 0.35	1.42 ± 0.53	0.818
BAS%	3.2 ± 0.2	2.9 ± 0.6	4.4 ± 0.8	0.147
PLT (10^3^/µL)	732.0 ± 96.7	596.0 ± 252.0	660.5 ± 168.3	0.811
MPV (fL)	6.60 ± 0.36	7.80 ± 0.68	6.80 ± 0.30	0.238
PCT%	0.48 ± 0.07	0.49 ± 0.21	0.44 ± 0.11	0.974
PDW	13.28 ± 1.42	14.43 ± 0.63	16.22 ± 3.64	0.614

Data are means ± SEM. The ANOVA testwasused to compare results between groups for normal distribution variables, and the Kruskal Wallis test for non-normal distribution variables. The Bonferroni post hoc test was used for multiple comparisons among groups. *P* < 0.05 indicates statistically significant differences with respect to their corresponding control group. Different superscript letters indicate significant differences between groups (a, b). One-way analysis of variance, ANOVA. Basophils, BAS. Eosinophils, EOS. Lymphocytes, LYM. Mean corpuscular hemoglobin, MCH. Mean corpuscular hemoglobin concentration, MCHC. Mean corpuscular volume. MCV. Monocyte, MON. Mean platelet volume, MPV. Total blood volume occupied by platelets, PCT. The percentage of total blood volume occupied by red blood cells packed red cell volume, PCT. Platelet distribution width, PDW. Platelet total count, PLT. Red blood cell count, RBC. Red blood cell size distribution, RDW. White blood cell count, WBC (count and percentages). Virgin olive oil, VOO.

**Table 2:** Blood hematological parameters after 90 days of intervention with different doses of the VOO extract.

	Control	100 mg (Low Dose)	300 mg (Intermediate Dose)	1000 mg (High Dose)	*P* Value
RBC (10^6^/µL)	7.51 ± 0.32 ^ab^	6.22 ± 0.42 ^a^	7.59 ± 0.25 ^ab^	7.99 ± 0.49 ^b^	0.010
Hemoglobin (g/dL)	14.0 ± 0.5	12.1 ± 0.8	14.3 ± 0.5	13.2 ± 0.7	0.071
PCV%	41.4 ± 1.6 ^ab^	33.9 ± 2.3 ^a^	41.0 ± 1.4 ^ab^	44.7 ± 3.1 ^b^	0.010
MCV (fl)	54.64 ± 0.50 ^ab^	54.71 ± 0.63 ^a^	54.0 ± 0.38 ^ab^	56.8 ± 0.80 ^b^	0.010
MCH	18.85 ± 0.22 ^a^	19.84 ± 0.71 ^ab^	18.79 ± 0.26 ^a^	16.17 ± 0.96 ^b^	0.001
MCHC (g Hb/dL)	34.50 ± 0.305 ^a^	36.18 ± 1.00 ^a^	34.79 ± 0.34 ^a^	28.85 ± 1.86 ^b^	0.001
RDW	14.04 ± 0.12	14.92 ± 0.62	13.79 ± 0.14	14.77 ± 0.21	0.055
WBC (10^6^/µL)	4.85 ± 0.46	3.19 ± 0.37	4.44 ± 0.51	4.21 ± 0.44	0.063
LYM (10^6^/µL)	3.80 ± 0.36	2.54 ± 0.40	3.42 ± 0.50	3.60 ± 0.40	0.156
MON (10^6^/µL)	0.06 ± 0.02	0.08 ± 0.03	0.07 ± 0.01	0.10 ± 0.02	0.706
NEU (10^6^/µL)	0.76 ± 0.09	0.51 ± 0.09	0.63 ± 0.09	0.48 ± 0.08	0.094
EOS (10^6^/µL)	0.05 ± 0.02	0.09 ± 0.03	0.16 ± 0.01	0.10 ± 0.04	0.636
BAS(10^6^/µL)	0.13 ± 0.01	0.08 ± 0.02	0.12 ± 0.02	0.09 ± 0.02	0.083
LYM%	78.0 ± 1.6	73.8 ± 2.8	75.9 ± 4.1	84.1 ± 1.4	0.055
MON%	1.4 ± 0.3	2.3 ± 0.7	1.5 ± 0.2	2.9 ± 0.7	0.145
NEU%	16.0 ± 1.2 ^a^	16.60 ± 2.09 ^a^	15.51 ± 1.82 ^a^	8.26 ± 1.28 ^b^	0.001
EOS%	1.5 ± 0.5	3.86 ± 1.20	3.99 ± 2.52	3.1 ± 1.00	0.634
BAS%	3.0 ± 0.3	3.45 ± 0.50	3.08 ± 0.33	2.66 ± 0.26	0.506
PLT (10^3^/µL)	684.5 ± 70.9 ^a^	399.1 ± 68.3 ^b^	541.7 ± 63.8 ^ab^	424.8 ± 72.2 ^ab^	0.016
MPV (fL)	6.13 ± 0.06 ^a^	7.23 ± 0.38 ^bc^	6.43 ± 0.18 ^ab^	7.19 ± 0.30 ^c^	0.001
PCT%	0.38 ± 0.034	0.26 ± 0.04	0.33 ± 0.04	0.28 ± 0.05	0.120
PDW	12.02±1.02	13.20 ± 1.03	13.56 ± 1.20	12.70 ± 1.56	0.825

Data are means ± SEM (*n* = 20). The ANOVA test was used to compare results between groups for normal distribution variables, and the Kruskal Wallis test for non-normal distribution variables. The Bonferroni post hoc test was used for multiple comparisons among groups. *P* < 0.05 indicates statistically significant differences with respect to their corresponding control group. Different superscript letters indicate significant differences between groups (a, b, c). One-way analysis of variance, ANOVA. Basophils, BAS. Eosinophils, EOS. Lymphocytes, LYM. Mean corpuscular hemoglobin, MCH. Mean corpuscular hemoglobin concentration, MCHC. Mean corpuscular volume, MCV. Monocytes, MON. Mean platelet volume, MPV. Differentiated neutrophils, NEU. The percentage of total blood volume occupied by platelets, PCT. The percentage of total blood volume occupied by red blood cells packed red cell volume, PCT. Platelet distribution width, PDW. Platelet total count, PLT. Total red blood cell count, RBC. Red blood cell size distribution, RDW. White blood cell count, WBC (count and percentages). Virgin olive oil, VOO.

**Table 3:** Plasma biochemical parameters after 14 days of intervention with different doses of the VOO extract.

	Control	300 mg (Low Dose)	2000 mg (Intermediate Dose)	*p* Value
Glucose (mg/dL)	149.91 ± 5.27	135.07 ± 11.73	145.11 ± 9.15	0.452
Cholesterol (mg/dL)	64.54 ± 2.87	64.39 ± 7.01	77.89 ± 3.09	0.062
Triacylglycerides (mg/dL)	99.12 ± 13.97	101.82 ± 22.55	69.72 ± 13.70	0.424
Proteins (g/dL)	5.72 ± 0.11	5.60 ± 0.10	5.86 ± 0.15	0.554
Albumin (g/dL)	3.05 ± 0.03	2.93 ± 0.06	3.04 ± 0.04	0.328
AST (U/L)	89.74 ± 6.13	91.19 ± 9.57	81.17 ± 7.56	0.677
ALT (U/L)	35.59 ± 2.44	32.06 ± 0.86	31.19 ± 1.83	0.433
Bilirrubin (mg/dL)	0.15 ± 0.03	0.14 ± 0.02	0.12 ± 0.01	0.859
Urea (mg/kg/dL)	27.7 ± 1.6	26.9 ± 4.06	29.6 ± 2.5	0.783
Creatinine (mg/kg/dL)	0.12 ± 0.02	0.08 ± 0.03	0.05 ± 0.02	0.212
Sodium (mg/kg/dL)	232.73 ± 5.79	242.77 ± 17.33	250.99 ± 14.50	0.417
Potassium (mg/kg/dL)	123.55 ± 21.35	210.04 ± 27.07	134.44 ± 41.81	0.186
Chloride (mg/kg/dL)	310.5 ± 2.0	300.0 ± 2.3	303.1 ± 6.4	0.119
Phosphorus (mg/dL)	3.8 ± 0.13	3.9 ± 0.17	4.1 ± 0.18	0.584
CK-MB (U/L)	374.6 ± 36.3 ^a^	549.8 ± 22.14 ^b^	418.0 ± 29.8 ^ab^	0.041

Data are means ± SEM. The ANOVA test wasused to compare results between groups for normal distribution variables, and the Kruskal Wallis test for non-normal distribution variables. The Bonferroni post hoc test was used for multiple comparisons among groups. *P* < 0.05 indicates statistically significant differences with respect to their corresponding control group. Different superscript letters indicate significant differences between post-intervention results (a, b). ALT, alanine amino-transaminase. One-way analysis of variance, ANOVA. Aspartate aminotransaminase, AST. Creatinin kinase isoenzyme MB, CK-MB. Standard error of the mean, SEM. Virgin olive oil, VOO.

**Table 4:** Plasma biochemical parameters after 90 days of intervention with different doses of the VOO extract.

	Control	100 mg (Low Dose)	300 mg (Intermediate Dose)	1000 mg (High Dose)	*p* Value
Glucose (mg/dL)	180.99 ± 9.58 ^ab^	154.38 ± 6.74 ^ab^	151.23 ± 10.52 ^a^	188.20 ± 11.65 ^b^	0.018
Cholesterol (mg/dL)	73.83 ± 3.20	76.34 ± 2.98	65.70 ± 4.29	76.12 ± 3.77	0.137
Triglycerides (mg/dL)	105.1 ± 10.3	103.9 ± 7.7	101.3 ± 8.5	122.3 ± 9.6	0.357
Proteins (g/dL)	6.36 ± 0.06 ^a^	6.05 ± 0.11 ^ab^	5.88 ± 0.17 ^b^	6.25 ± 0.10 ^ab^	0.024
Albumin (g/dL)	3.2 ± 0.02	3.2 ± 0.03	3.2 ± 0.07	3.4 ± 0.115	0.295
Albumin/Globulin	1.0 ± 0.5	1.0 ± 0.3	1.1 ± 0.7	1.2 ± 0.3	0.159
AST (U/L)	96.20 ± 8.73	87.38 ± 17.18	68.08 ± 6.38	92.11 ± 12.90	0.368
ALT (U/L)	43.03 ± 3.74	33.33 ± 1.93	34.41 ± 2.85	37.04 ± 3.31	0.118
Bilirubin (mg/dL)	0.69 ± 0.005 ^a^	0.74 ± 0.005 ^ab^	0.77 ± 0.004 ^b^	0.70 ± 0.024 ^a^	<0.001
Urea (mg/kg/dL)	45.8 ± 1.4	41.1 ± 1.5	43.2 ± 1.9	43.4 ± 1.7	0.289
Creatinine (mg/kg/dL)	0.32 ± 0.02	0.26 ± 0.02	0.27 ± 0.03	0.25±0.02	0.067
Sodium (mg/kg/dL)	183.60 ± 4.52 ^a^	237.14 ± 8.64 ^b^	214.06 ± 7.75 ^b^	226.28 ± 8.16 ^b^	0.001
Potassium (mg/kg/dL)	105.69 ± 8.34	116.67 ± 8.78	119.31 ± 10.15	114.95 ± 10.54	0.759
Chloride (mg/kg/dL)	366.6 ± 7.9	334.4 ± 3.1	342.5 ± 17.8	337.5 ± 2.8	0.090
Phosphorus (mg/dL)	5.7 ± 0.0.21	4.9 ± 0.21	6.7 ± 0.93	5.8 ± 0.24	0.095
CK-MB (U/L)	463.5 ± 58.2	376.5 ± 69.3	308.0 ± 34.0	410.9 ± 52.2	0.250

Data are means ± SEM (*n* = 20), except in the 100 mg and 300 mg group because defective lysis samples were dismissed. The ANOVA test wasused to compare results between groups for normal distribution variables, and the Kruskal Wallis test was used for non-normal distribution variables. The Bonferroni post hoc test was used for multiple comparisons among groups. *P* < 0.05 indicates statistically significant differences with respect to their corresponding control group. Different superscript letters indicate significant differences between post-intervention results (a, b,). Alanine amino-transaminase, ALT. One-way analysis of variance, ANOVA. Aspartate aminotransaminase, AST. Creatinin kinase isoenzyme MB, CK-MB. Standard error of the mean, SEM. Virgin olive oil, VOO.
